# Complete genome sequences of *Geobacillus* sp. Y412MC52, a xylan-degrading strain isolated from obsidian hot spring in Yellowstone National Park

**DOI:** 10.1186/s40793-015-0075-0

**Published:** 2015-10-19

**Authors:** Phillip Brumm, Miriam L. Land, Loren J. Hauser, Cynthia D. Jeffries, Yun-Juan Chang, David A. Mead

**Affiliations:** C5•6 Technologies Inc., Middleton, WI USA; Oak Ridge National Laboratory, Oak Ridge, TN USA; Bioscience Division, Los Alamos National Laboratory, Los Alamos, NM USA; Lucigen Corporation, Middleton, WI USA

**Keywords:** *Geobacillus* sp. Y412MC52, Obsidian hot spring, Biomass, Arabinan, Xylan, *G. thermocatenulatus*

## Abstract

*Geobacillus* sp. Y412MC52 was isolated from Obsidian Hot Spring, Yellowstone National Park, Montana, USA under permit from the National Park Service. The genome was sequenced, assembled, and annotated by the DOE Joint Genome Institute and deposited at the NCBI in December 2011 (CP002835). Based on 16S rRNA genes and average nucleotide identity, *Geobacillus sp.* Y412MC52 and the related *Geobacillus sp.* Y412MC61 appear to be members of a new species of *Geobacillus*. The genome of *Geobacillus* sp. Y412MC52 consists of one circular chromosome of 3,628,883 bp, an average G + C content of 52 % and one circular plasmid of 45,057 bp and an average G + C content of 45 %. Y412MC52 possesses arabinan, arabinoglucuronoxylan, and aromatic acid degradation clusters for degradation of hemicellulose from biomass. Transport and utilization clusters are also present for other carbohydrates including starch, cellobiose, and α- and β-galactooligosaccharides.

**Electronic supplementary material:**

The online version of this article (doi:10.1186/s40793-015-0075-0) contains supplementary material, which is available to authorized users.

## Introduction

Identification of new organisms that produce biomass-degrading enzymes is of considerable interest. Commercial uses for these enzymes include paper manufacturing, brewing, biomass deconstruction and the production of animal feeds [1–3]. Hot springs, especially those at Yellowstone National Park, have been a source of many new organisms including *Thermus aquaticus* [4, 5], *Thermus brockianus* [6], and *Acidothermus cellulolyticus* [7] that possess enzymes with significant potential in biotechnological applications [8]. As part of a project in conjunction with the Great Lakes Bioenergy Research Center, Dept. of Energy, C5–6 Technologies and Lucigen Corp. isolated, characterized, and sequenced a number of new enzyme-producing aerobic organisms from Yellowstone hot springs.

*Geobacillus* species were the most common aerobic organisms isolated during the cultivation of most hot springs samples. *Geobacillus* species were originally classified as members of the genus *Bacillus*, but were subsequently reclassified as a separate genus based on 16S rRNA gene sequence analysis, lipid and fatty acid analysis, phenotypic characterization, and DNA—DNA hybridization experiments [9]. *Geobacillus* species have been isolated from a number of extreme environments including high-temperature oilfields [10], a corroded pipeline in an extremely deep well [11], African [12] and Russian [13] hot springs, marine vents [14], and the Mariana Trench [15], yet they can also be found in garden soils [16] and hay composts [17]., The ability of *Geobacillus* species to thrive in these varied and often hostile environments suggests that these species possess enzymes suitable for applications in challenging industrial environments. We therefore sequenced a number of these *Geobacillus* isolates including strains Y41MC52, Y41MC61, C56-T3, and Y4.1MC1 [18] to identify new enzymes suitable for use in biomass conversion into fuels and chemicals.

### Organism information

#### Classification and features

**Fig. 1:**
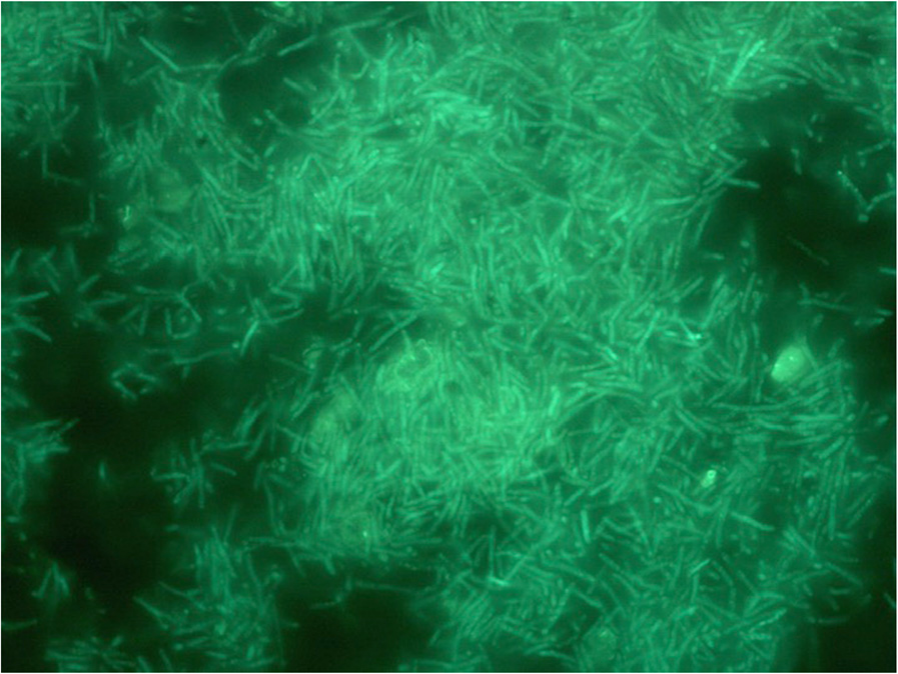
Micrograph of *Geobacillus* sp. Y412MC52 cells showing individual cells and clumps of cells. Cells were grown in TSB plus 0.4 % glucose for 18 h. at 70 °C. A 1.0 ml aliquot was removed, centrifuged, re-suspended in 0.2 ml of sterile water, and stained using a 50 μM solution of SYTO^®^ 9 fluorescent stain in sterile water (Molecular Probes). Dark field fluorescence microscopy was performed using a Nikon Eclipse TE2000-S epifluorescence microscope at 2000× magnification using a high-pressure Hg light source and a 500 nm emission filter

*Geobacillus* sp. Y412MC52 and *Geobacillus* sp. Y412MC61 are two thermophilic organisms isolated from Obsidian Hot Spring, Yellowstone National Park, Montana, USA (44.6100594° latitude and −110.4388217° longitude) under a sampling permit from the National Park Service. The hot spring possesses a pH of 6.37 and a temperature range of 42–90 °C. The organisms were isolated from a sample of hot spring water by enrichment and plating on YTP-2 medium [19] at 70 °C. The cultures are available from the *Bacillus* Genetic Stock Center as GSCID: 96A11 (MC52) and GSCID: 96A12 (MC61). Both cultures are routinely grown in YTP-2 medium media and maintained on YTP-2 agar plates. MC52, is a Gram-positive, rod-shaped facultative anaerobe (Table 1 and Additional file 1: Table S1), with optimum growth temperature of 65 °C and maximum growth temperature of 75 °C. MC52 appears to grow as a mixture of single cells and occasional large clumps of cells in liquid culture (Fig. 1). Growth is not observed on minimal medium supplemented with glucose, xylose or other sugars. Excellent growth is seen in Luria Broth, Terrific Broth, Tryptic Soy Broth and other common lab media with and without additional carbohydrate, indicating potential growth requirements for both vitamins and amino acids. Growth in YTP-2 medium is stimulated by addition of monosaccharides, disaccharides, soluble starch, xylan, arabinan, and arabinogalactan. Growth in YTP-2 medium is not stimulated by addition of cellulose, mannan, glucomannan, galactomannan, chitin, or pectin. MC52 produces extracellular xylanase when grown in YTP-2 medium supplemented with pyruvate, xylose, xylooligosaccharides and arabinogalactan. No secreted xylanase is detected when MC52 is grown in YTP-2 medium supplemented with glucose or arabinose. Extracellular arabinase is detected only in cultures grown in YTP-2 medium supplemented with arabinogalactan. Extracellular amylase is detected in cultures grown in YTP-2 medium supplemented with soluble starch or pullulan. Blue (positive) colonies of MC52 are observed on plates containing either 5-bromo-4-chloro-3-indolyl-β-D-galactopyranoside or 5-bromo-4-chloro-3-indolyl-α-D-galactopyranoside, indicating production of α-galactosidase and β-galactosidase. Fluorescent colonies are observed on plates containing 4-methylumbelliferyl-β-D-cellobioside, 4-methylumbelliferyl-β-D-xylopyranoside, and 4-methylumbelliferyl-β-D-glucoyranoside indicating production of β-glucosidase and β-xylosidase.

**Table 1:** Classification and general features of *Geobacillus* sp. Y412MC52 [46]

MIGS ID	Property	Term	Evidence code^a^
	Classification	Domain *Bacteria*	TAS [47]
		Phylum *Firmicutes*	TAS [48, 49]
		Class *Bacilli*	TAS [48, 49]
		Order *Bacillales*	TAS [48, 49]
		Family *Bacillaceae*	TAS [48, 49]
		Genus *Geobacillus*	TAS [9, 49]
		Species	IDA
		Strain Y412MC52	IDA
	Gram stain	Positive	IDA
	Cell shape	Rods	IDA
	Motility	Motile	IDA
	Sporulation	Spore former	NAS
	Temperature range	55 to 75 °C	IDA
	Optimum temperature	65 °C	IDA
	pH range; Optimum	5.5–8.0; 7.5	IDA
	Carbon source	Monosaccharides, xylan, arabinan	IDA
MIGS-6	Habitat	Hot spring	IDA
MIGS-6.3	Salinity	Not reported	IDA
MIGS-22	Oxygen requirement	Facultative anaerobe	IDA
MIGS-15	Biotic relationship	Free-living	IDA
MIGS-14	Pathogenicity	Non-pathogen	NAS
MIGS-4	Geographic location	Obsidian spring, Yellowstone National Park	IDA
MIGS-5	Sample collection	September 2003	IDA
MIGS-4.1	Latitude	44.6603028	IDA
MIGS-4.2	Longitude	−110.865194	IDA
MIGS-4.4	Altitude	2416 m	IDA

^a^Evidence codes - IDA: Inferred from Direct Assay; TAS: Traceable Author Statement (i.e., a direct report exists in the literature); NAS: Non-traceable Author Statement (i.e., not directly observed for the living, isolated sample, but based on a generally accepted property for the species, or anecdotal evidence). These evidence codes are from the Gene Ontology project [50]

**Fig. 2:**
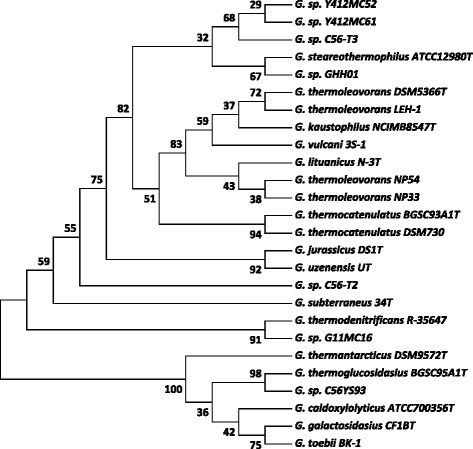
The evolutionary history was inferred by using the Maximum Likelihood method based on the Tamura-Nei model [22]. The bootstrap consensus tree inferred from 500 replicates [45] is taken to represent the evolutionary history of the taxa analyzed [45]. Branches corresponding to partitions reproduced in less than 50 % bootstrap replicates are collapsed. The percentage of replicate trees in which the associated taxa clustered together in the bootstrap test (500 replicates) are shown next to the branches [45]. Initial tree(s) for the heuristic search were obtained automatically by applying Neighbor-Join and BioNJ algorithms to a matrix of pairwise distances estimated using the Maximum Composite Likelihood (MCL) approach, and then selecting the topology with superior log likelihood value. The analysis involved 26 nucleotide sequences. All positions containing gaps and missing data were eliminated. There were a total of 1271 positions in the final dataset. Evolutionary analyses were conducted in MEGA5 [21]. The type strains of all validly described species are included (NCBI accession numbers): *G. caldoxylosilyticus* ATCC700356^T^ (AF067651), *G. galactosidasius* CF1B^T^ (AM408559), *G. jurassicus* DS1^T^ (FN428697), *G. kaustophilus* NCIMB8547^T^ (X60618), *G. lituanicus* N-3^T^ (AY044055), *G. stearothermophilus* R-35646^T^ (FN428694), *G. subterraneus* 34^T^ (AF276306), *G. thermantarcticus* DSM9572^T^ (FR749957), *G. thermocatenulatus* BGSC93A1^T^ (AY608935), *G. thermodenitrificans* R-35647^T^ (FN538993), *G. thermoglucosidasius* BGSC95A1^T^ (FN428685), *G. thermoleovorans* DSM5366^T^ (Z26923), *G. toebii* BK-1^T^ (FN428690), *G. uzenensis* U^T^ (AF276304) and *G. vulcani* 3S-1^T^ (AJ293805). Additional16S rRNA sequences of *G. thermoleovorans* strain NP54 (JN871595*G. thermoleovorans* strain NP33 (JQ343209), *G. thermoleovorans* strain LEH-1 (NR_036985), G. *thermocatenulatus* strain DSM 730 (NR_119305), *G. vulcani* 3S-1 (NR_025426), *G.* strain C56-T3 (NC_014206), *G.* strain GHH01 (NC_020210), *G.* strain C56-YS93 (CP002835), and *G.* strain G11MC16 (CP002835)

A phylogenetic tree was constructed to identify the relationship of *Geobacillus* sp. Y412MC52 and *Geobacillus* sp. Y412MC61 to other members of the *Geobacillus* family. MC52 and MC61 both contain eight annotated 16S rRNA genes. The 16S rRNA genes located at MC52 genome coordinates 11,820 through 13,365 and MC61 genome coordinates 10,516 through 12,061 were used for tree construction. Trees constructed with the remaining seven MC52 16S rRNA genes were identical to the tree shown here. The phylogeny was determined using the described 16S rRNA gene sequences, 16S rRNA gene sequences of the type strains of all validly described *Geobacillus* species and full-length 16S rRNA gene sequences of *Geobacillus* species present in GenBank. The 16S rRNA gene sequences were aligned using MUSCLE [20], pairwise distances were estimated using the Maximum Composite Likelihood approach, and initial trees for heuristic search were obtained automatically by applying the Neighbour-Joining method in MEGA 5 [21]. The alignment and heuristic trees were then used to infer the phylogeny using the Maximum Likelihood method based on Tamura-Nei [22]. The phylogenetic tree (Fig. 2) indicates that MC52, MC61 and *Geobacillus* sp. C56-T3 cluster separately from other validly named species.

### Genome sequencing and annotation

#### Genome project history

Y412MC52 was selected for sequencing on the basis of its biotechnological potential as part of the U.S. Department of Energy Genomic Science program (formerly Genomics:GTL). The genome sequence is deposited in the Genomes On Line Database [23, 24] (GOLD ID = Gc01757), and in GenBank (NCBI Reference Sequence = CP002442.1). Sequencing, finishing and annotation were performed by the DOE Joint Genome Institute. A summary of the project information and its association with MIGS identifiers is shown in Table 2.

**Table 2:** Project information

MIGS ID	Property	Term
MIGS 31	Finishing quality	Finished
MIGS-28	Libraries used	6 kb and 24 kb
MIGS 29	Sequencing platforms	454 Titanium, Illumina GAii
MIGS 31.2	Fold coverage	5.8
MIGS 30	Assemblers	Phred/Phrap/Consed
MIGS 32	Gene calling method	Prodigal, GenePRIMP
	Locus tag	GYMC52
	Genbank ID	CP002835.1
	GenBank date of release	July 1, 2011
	GOLD ID	Gc01757
	BIOPROJECT	PRJNA30797
MIGS 13	Source material identifier	BGSCID: 96A11
	Project relevance	Biotechnological

### Growth conditions and genomic DNA preparation

For preparation of genomic DNA, cultures of Y51MC23 were grown from a single colony in YTP-2 in 1000 ml medium in a 2000 ml Erlenmeyer flask at 70 °C, 200 rpm for 18 h. Cells were collected by centrifugation at 4 °C and stored frozen until used for DNA preparation. The cell concentrate was lysed using a combination of SDS and proteinase K, and genomic DNA was isolated using a phenol/chloroform extraction method [25]. The genomic DNA was precipitated, and treated with RNase to remove residual contaminating RNA.

### Genome sequencing and assembly

The genome of *Geobacillus* sp. Y412MC52 was sequenced at the Joint Genome Institute (JGI) using a combination of Sanger, Illumina and 454 technologies [26]. An Illumina GAii shotgun library with reads of 664 Mb, a 454 Titanium draft library with average read length of 250 bp, and two Sanger libraries with average insert size of 3 and 8 Kb were generated for this genome. Illumina sequencing data was assembled with VELVET [27], and the consensus sequences were shredded into 1.5 Kb overlapped fake reads and assembled together with the 454 data. Draft assemblies were based on 95.5 MB 454 draft data. Newbler parameters are - consed -a 50–1 350 -g -m -ml 20. The initial Newbler assembly contained 40 contigs in 18 scaffolds. We converted the initial 454 assembly into a phrap assembly by making fake reads from the consensus, collecting the read pairs in the 454 paired end library. The Phred/Phrap/Consed software package was used for sequence assembly and quality assessment [28–30] in the following finishing process. Illumina data was used to correct potential base errors and increase consensus quality using a software Polisher developed at JGI (Alla Lapidus, unpublished). After the shotgun stage, reads were assembled with parallel phrap (High Performance Software, LLC). Possible mis-assemblies were corrected with gapResolutioin (Cliff Han, unpublished), Dupfinisher, or sequencing cloned bridging PCR fragments with subcloning. Gaps between contigs were closed by editing in Consed, by PCR and by Bubble PCR primer walks. A total of 1069 additional reactions and 9 shatter libraries were necessary to close gaps and to raise the quality of the finished sequence. The overall average error rate achieved was 0.01 errors/10 Kb.

### Genome annotation

Genes were identified using Prodigal [31] as part of the Oak Ridge National Laboratory genome annotation pipeline, followed by a round of manual curation using the JGI GenePRIMP pipeline [32]. The predicted CDSs were translated and used to search the National Center for Biotechnology Information (NCBI) nonredundant database, UniProt, TIGRFam, Pfam, PRIAM, KEGG, COG, and InterPro databases. These data sources were combined to assert a product description for each predicted protein. Non-coding genes and miscellaneous features were predicted using tRNAscan-SE [32], RNAMMer [33], Rfam [34], TMHMM [35], and signalP [35].

### Genome properties

**Fig. 3:**
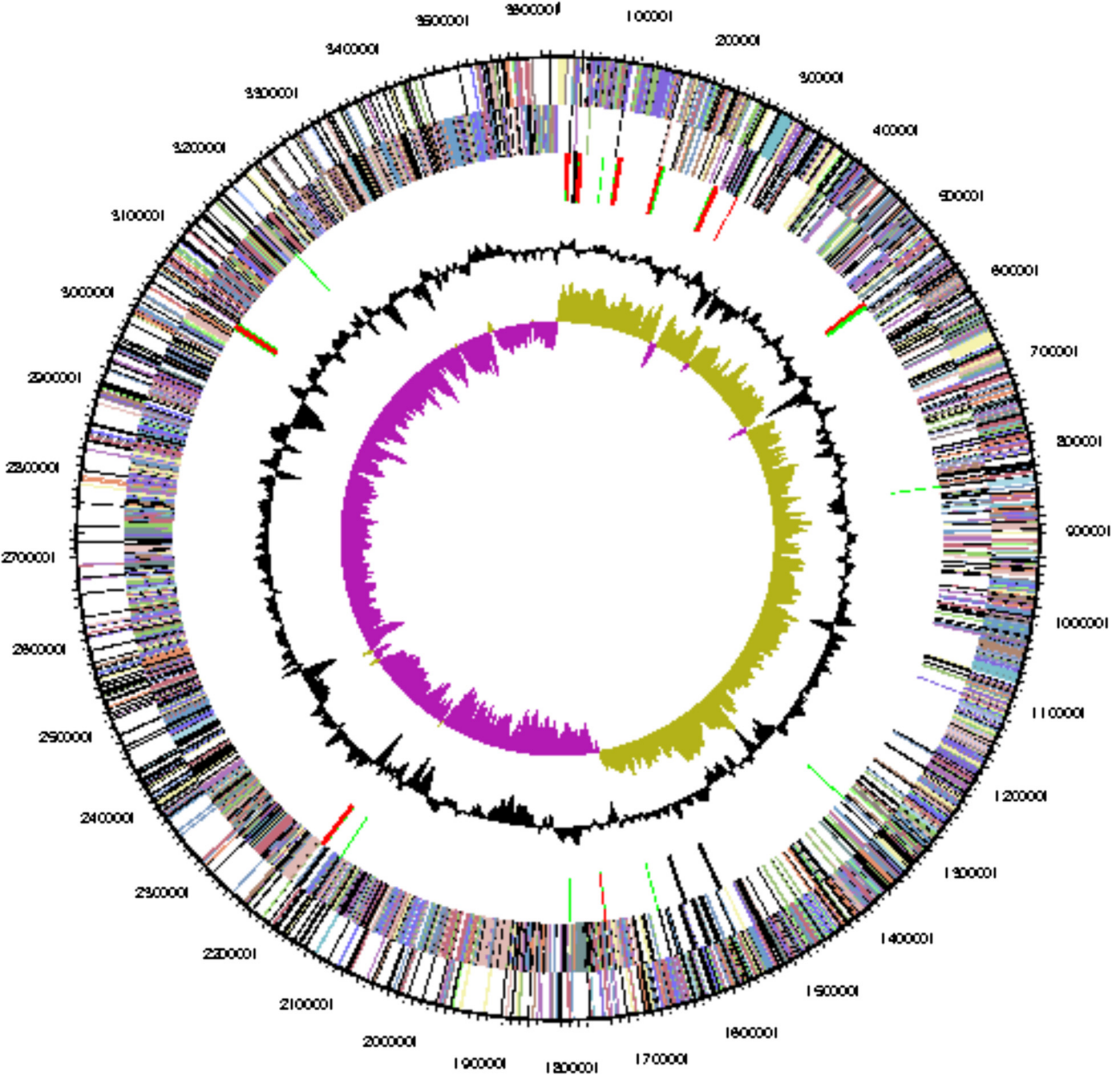
Graphical circular map of the Y412MC52 chromosome. From outside to the center: Genes on forward strand (color by COG categories) Genes on reverse strand (color by COG categories) RNA genes (tRNAs green, rRNAs red, other RNAs black) GC content, GC skew

The genome of *Geobacillus* sp. Y412MC52 consists of one circular chromosome of 3,628,883 bp (Table 3 and Fig. 3) and an average G + C content of 52 % and one circular plasmid of 45,057 bp and an average G + C content of 45 % (Table 4). There are 88 tRNA genes, 25 rRNA genes and 3 “other” identified RNA genes. There are 3634 predicted protein-coding regions and 175 pseudogenes in the genome. A total of 2569 genes (68.51 %) have been assigned a predicted function while the rest have been designated as hypothetical proteins (Table 4). The numbers of genes assigned to each COG functional category are listed in Table 5. About 35 % of the annotated genes were not assigned to a COG or have an unknown function.

**Table 3:** Summary of genome: 1 chromosome and 1 plasmid

Label	Size (Mb)	Topology	INSDC identifier	RefSeq ID
Chromosome	3.62	Circular	CP002442	NC_014915
Plasmid 1	0.045	Circular	CP002443	NC_014916

**Table 4:** Genome statistics

Attribute	Value
Genome size (bp)	3,673,940
DNA coding (bp)	3,199,671
DNA G + C (bp)	1,922,887
DNA scaffolds	2
Total genes	3750
Protein-coding genes	3634
RNA genes	116
Pseudo genes	175
Genes in internal clusters	1984
Genes with function prediction	2569
Genes assigned to COGs	2414
Genes with Pfam domains	3048
Genes with signal peptides	174
Genes with transmembrane helices	873
CRISPR repeats	6

**Table 5:** Number of genes associated with general COG functional categories

Code	Value	Percent	Description
J	149	5.59	Translation, ribosomal structure and biogenesis
A	0	0	RNA processing and modification
K	180	6.76	Transcription
L	156	5.86	Replication, recombination and repair
B	1	0.04	Chromatin structure and dynamics
D	31	1.16	Cell cycle control, cell division, chromosome partitioning
V	36	1.35	Defense mechanisms
T	124	4.65	Signal transduction mechanisms
M	104	3.90	Cell wall/membrane/envelope biogenesis
N	58	2.18	Cell motility
U	46	1.73	Intracellular trafficking, secretion, and vesicular transport
O	81	3.04	Posttranslational modification, protein turnover, chaperones
C	157	5.89	Energy production and conversion
G	193	7.24	Carbohydrate transport and metabolism
E	258	9.68	Amino acid transport and metabolism
F	71	2.07	Nucleotide transport and metabolism
H	126	4.73	Coenzyme transport and metabolism
I	118	4.43	Lipid transport and metabolism
P	121	4.54	Inorganic ion transport and metabolism
Q	70	2.63	Secondary metabolites biosynthesis, transport and catabolism
R	304	11.41	General function prediction only
S	280	10.51	Function unknown
	1336	35.63	Not in COGs

The total is based on the total number of protein coding genes in the annotated genome

### Insights from the genome sequence

**Fig. 4:**
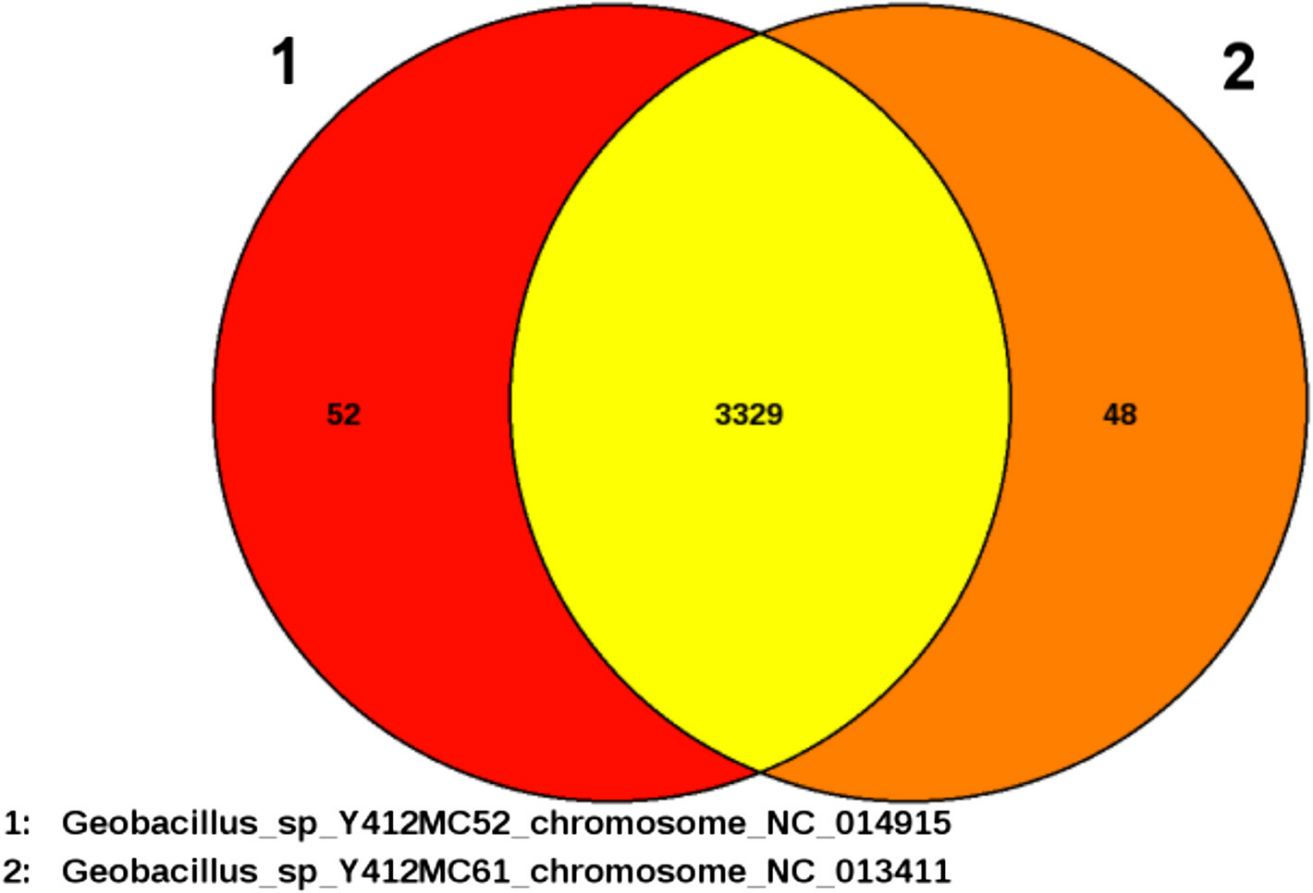
Venn Diagram of Y412MC52 and Y412MC61 determined using software at https://edgar.computational.bio.uni-giessen.de

**Fig. 5:**
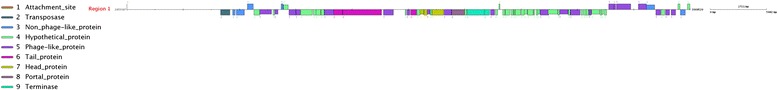
Prophage insert in Y412MC52 identified using phast [41, 42]

Average Nucleotide Identity (ANI) calculations [36] were used to compare the genomes of MC52 and other sequenced *Geobacillus* species. The comparison of the MC52 genome to the other genomes (Table 6) confirms the phylogenetic tree obtained using 16S rRNA genes. MC52 is most closely related to MC61 (100 % identity) followed by *Geobacillus* sp. C56-T3 (98.3 %). These values are above the species cutoff value of 98.2 % to 99.0 % [37] indicating that these are most likely strains of the same species. The ANI values for all other sequenced strains are below 98 %, suggesting that MC52, MC61, and C56-T3 represent members of a new species. Comparison of genes shows MC52 and MC61 share 3329 genes (Fig. 4). MC52 has 52 unique genes and MC61 has 48. These unique genes code mostly for hypothetical proteins and are randomly distributed throughout both genomes. Alignment of the MC52 and M61 genomes using progressiveMauve [38] shows one predominant, four medium, and two small Locally Collinear Blocks of conserved genes (Fig. 5). In Y412MC61, two of the medium blocks precede the predominant block, while these blocks follow the predominant block in Y412MC52. In addition to having alternate locations within these genomes, these two blocks reverse their orientation between the two genomes. Taken together, these results indicate that MC52 and M61 are not two different isolates of the same strain, but are two closely related strains of the same species with a unique relationship to each other.

**Table 6:** Average Nucleotide Identity with MC52

Strain	ANI
*Geobacillus sp.* Y412MC61	100
*Geobacillus sp.* C56-T3	98.3
*Geobacillus sp.* CAMR12739	97.6
*Geobacillus sp.* MAS1	96.9
*G. kaustophilus* HTA426	96.7
*Geobacillus sp.* A8	96.7
*G. thermoleovorans* CCB_US3_UF5	96.7
*G. thermoleovorans* B23	96.7
*Geobacillus sp.* FW23	96.7
*G. kaustophilus* GBlys	96.6
*Geobacillus sp.* GHH01	96.5
*G. kaustophilus* NBRC 102445	96.4
*Geobacillus sp.* WSUCF1	96.2
*Geobacillus sp.* CAMR5420	96.1
*G. thermocatenulatus* GS-1	94.7
*G. vulcani* PSS1	91.3
*G. stearothermophilus* 22	89.6

Values obtained from IMG database [51]

MC52 possesses a 45-gene arabinan and xylan degradation cluster that allows degradation of hemicellulose components of biomass (GYMC52_1817 through GYMC52_1867). The cluster contains one secreted xylanase (GYMC52_1825) and one secreted arabinase (GYMC52_1858), in agreement with the experimental results. The organization of the xylan degradation portion of the cluster matches the glucuronic acid utilization cluster described for *G. stearothermophilus* [39]. The arabinan degradation part of the cluster is smaller than the arabinan cluster of *G. stearothermophilus* [40], lacking araP, araS, araT, araE, araG and araH genes. MC52 also possesses three clusters annotated for degradation of aromatic acid molecules, GYMC52_1956 through GYMC52_1962, GYMC52_1990 through GYMC52_2001, and GYMC52_3134 through GYMC52_3141. Geobacillus species utilize xylan by transporting large xylooligosaccharides into the cell and then degrading these xylooligosaccharides intracellularly [39]. These aromatic acid degradation clusters may allow degradation and utilization of lignin fragments such as ferulic, sinapic, and cinnamic acids that are attached to the xylooligosaccharides. Utilization of these aromatic acids increases the metabolic energy obtained from the fragments and eliminates potential toxicity of these aromatic acids. Transport and metabolic clusters for utilization of cellobiose and related oligosaccharides (GYMC52_1797 through GYMC52_1801), α- and β-galactooligosaccharides (GYMC52_12121 through GYMC52_2132), and α-1,4-linked glucooligosaccharides (GYMC52_06321 through GYMC52_0637) were identified, confirming the experimental observations of the corresponding enzymatic activities.

The smaller arabinan cluster in MC52 is the result of an 11-gene insert (GYMC52_1870 through GYMC52_1880) coding for a peptide utilization cluster that replaces part of the arabinan cluster. This peptide utilization cluster is found in only a few *Geobacillus* strains, including *Geobacillus sp.* Y412MC61 (GYMC61_2740 through GYMC52_2750), *Geobacillus sp.* Y4.1MC1 (GY4MC1_2192 through GY4MC1_2202), and *Geobacillus sp.* C56-YS93 (Geoth_2276 through Geoth_2288). The cluster does not code for a secreted protease or peptidase, but contains an annotated five-gene ABC peptide transporter system and two intracellular peptidases.

*Geobacillus* strain Y412MC52 possesses a 54.4 Kb, 73-gene insert that codes for 47 phage genes identified using phast [41, 42] phage identification software (Fig. 5), an identical insert is present in Y412MC61. The prophage insert has 39 % coverage and 83 % identity to *Geobacillus* phage E2 (GenBank NC_009552) [43], isolated from a deep sea location. The phage is not present in *Geobacillus* strain C56-YS93 also isolated from Obsidian Hot Spring, indicating the phage may have a limited range of hosts in the hot spring.

## Conclusions

Obsidian Hot Spring is home to a wide variety of organisms, including *Paenibacillus lautus* Y412MC10 [19], *Geobacillus thermoglucosidans* C56-YS93 (manuscript submitted) and *Geobacillus sp.* Y412MC52 and Y412MC61. Especially of interest is the isolation of both low G + C (C56-YS93, 43.9 % G + C) and high G + C (Y412MC52 and Y412MC61, 52.3 % G + C) xylanolytic *Geobacillus* species from the same hot spring sample. This suggests that the high and low G + C *Geobacillus* species may occupy separate ecological niches that allow each strain to thrive in the same site. Based on the genomic analysis, *Geobacillus sp.* Y412MC52 appears to utilize only some biomass components such as xylan, arabinoglucuronoxylan, and the arabinan component of arabinogalactan. MC52 shows no genes coding for utilization of other biomass components such as cellulose, mannan, glucomannan, galactomannan, chitin, or pectin, confirming experimental observations. The limited range of substrates suggests MC52 functions as part of a microbial consortium in degrading biomass. The presence of aromatic acid metabolic clusters and the lack of mannan-utilization clusters suggest the organism has a preference for utilization of hemicellulose derived from grassy plants rather than woody plants.

Based on 16S rRNA genes and average nucleotide identity, *Geobacillus sp.* Y412MC52 and the related *Geobacillus sp.* Y412MC61 appear to be members of a new species of *Geobacillus*. The presence of multiple 16S rRNA genes in *Geobacillus* species as well as the small differences observed in 16S rRNA gene sequences makes assignment of strains to new or existing species difficult. Utilization of recN sequences [44] has been proposed as an alternative to 16S rRNA gene sequences, but it is unclear if this leads to a more accurate description of the distinct species. Sequencing of additional genomes and in-depth microbiological characterizations are needed to clarify the relationships among *Geobacillus* species.

## Additional file

Additional file 1: Table S1. Associated MIGS record. (DOCX 33 kb)

## Abbreviations

MC52: *Geobacillus* sp. Y412MC52
MC61: *Geobacillus* sp. Y412MC61
