# Erratum to: Complete genome sequences of Geobacillus sp. Y412MC52, a xylan-degrading strain isolated from obsidian hot spring in Yellowstone National Park

**DOI:** 10.1186/s40793-016-0133-2

**Published:** 2016-01-26

**Authors:** Phillip Brumm, Miriam L. Land, Loren J. Hauser, Cynthia D. Jeffries, Yun-Juan Chang, David A. Mead

**Affiliations:** C5•6 Technologies Inc, Middleton, WI USA; Oak Ridge National Laboratory, Oak Ridge, TN USA; Bioscience Division, Los Alamos National Laboratory, Los Alamos, NM USA; Lucigen Corporation, Middleton, WI USA

After publication of this study [1], the authors noticed that GenBank accession number is incorrect. The correct GenBank accession number is CP002442.
