# Supplementary material for: Complete genome sequences of Geobacillus sp. Y412MC52, a xylan-degrading strain isolated from obsidian hot spring in Yellowstone National Park
Source: Stand Genomic Sci. 2015 Oct 19;10:81. doi: 10.1186/s40793-015-0075-0 (PMC4617443; doi:10.1186/s40793-015-0075-0)
Supplement: Additional file 1: Table S1. — Associated MIGS record. (DOCX 33 kb) [file 40793_2015_75_MOESM1_ESM.docx]

**Additional file 1: Table S1.** Associated MIGS record.

| **MIGS-ID** | field name | Description |
| --- | --- | --- |
| **MIGS-1** | Submit to INSDC/Trace archives |  |
| **1.1** | PID |  |
| **1.2** | Trace Archive |  |
| **MIGS-2** | MIGS CHECK LIST TYPE |  |
| **MIGS-3** | Project Name |  |
| **MIGS-4** | Geographic Location | Yellowstone National Park |
| **4.1** | Latitude | 44.6100594 |
| **4.2** | Longitude | -110.4388217 |
| **4.3** | Depth | Surface |
| **4.4** | Altitude | 2416 m |
| **MIGS-5** | Time of Sample collection | September 2003 |
| **MIGS-6** | Habitat (EnvO) | Hot spring |
| **6.1** | Temperature | 79±4°C |
| **6.2** | pH | 6.7±0.4 |
| **6.3** | Salinity |  |
| **6.4** | Chlorophyll |  |
| **6.5** | Conductivity | 324-1076 |
|  |  |  |
| **6.6** | light intensity |  |
| **6.7** | dissolved organic carbon (DOC) |  |
| **6.8** | Current |  |
| **6.9** | atmospheric data |  |
| **6.10** | Density |  |
| **6.11** | Alkalinity | 134 |
| **6.12** | dissolved oxygen | 69.1 |
| **6.13** | particulate organic carbon (POC) |  |
| **6.14** | Phosphate |  |
| **6.15** | Nitrate | 31 |
| **6.16** | Sulfates | 294-702 |
| **6.17** | Sulfides |  |
| **6.18** | primary production |  |
| **MIGS-7** | Subspecific genetic lineage |  |
| **MIGS-9** | Number of replicons |  |
| **MIGS-10** | Extrachromosomal elements |  |
| **MIGS-11** | Estimated Size |  |
| **MIGS-12** | Reference for biomaterial or Genome report |  |
| **MIGS-13** | Source material identifiers |  |
| **MIGS-14** | Known Pathogenicity | none |
|  |  |  |
| **MIGS-15** | Biotic Relationship | free-living |
| **MIGS-16** | Specific Host |  |
| **MIGS-17** | Host specificity or range (taxid) |  |
| **MIGS-18** | Health status of Host |  |
| **MIGS-19** | Trophic Level |  |
| **MIGS-22** | Relationship to Oxygen | facultative anaerobe |
| **MIGS-23** | Isolation and Growth conditions |  |
| **MIGS-27** | Nucleic acid preparation |  |
| **MIGS-28** | Library construction |  |
| **28.1** | Library size |  |
| **28.2** | Number of reads |  |
| **28.3** | Vector |  |
| **MIGS-29** | Sequencing method |  |
| **MIGS-30** | Assembly |  |
| **30.1** | Assembly method |  |
| **30.2** | estimated error rate |  |
| **30.3** | method of calculation |  |
| **MIGS-31** | Finishing strategy |  |
| **31.1** | Status |  |
| **31.2** | Coverage |  |
| **31.3** | Contigs |  |
| **MIGS-32** | Relevant SOPs |  |
| **MIGS-33** | Relevant e-resources |  |
